# Epigenetic control in Kaposi sarcoma-associated herpesvirus infection and associated disease

**DOI:** 10.1007/s00281-020-00787-z

**Published:** 2020-03-26

**Authors:** Jacqueline Fröhlich, Adam Grundhoff

**Affiliations:** Heinrich-Pette-Institute, Leibniz Institute for Experimental Virology, Hamburg, Germany

**Keywords:** KSHV, Latency, Tumor virus, Epigenetic modifications, Chromatin, DNA methylation

## Abstract

Kaposi sarcoma-associated herpesvirus (KSHV) is the etiologic agent of several malignancies of endothelial and B-cell origin. The fact that latently infected tumor cells in these malignancies do not express classical viral oncogenes suggests that pathogenesis of KSHV-associated disease results from multistep processes that, in addition to constitutive viral gene expression, may require accumulation of cellular alterations. Heritable changes of the epigenome have emerged as an important co-factor that contributes to the pathogenesis of many non-viral cancers. Since KSHV encodes a number of factors that directly or indirectly manipulate host cell chromatin, it is an intriguing possibility that epigenetic reprogramming also contributes to the pathogenesis of KSHV-associated tumors. The fact that heritable histone modifications have also been shown to regulate viral gene expression programs in KSHV-infected tumor cells underlines the importance of epigenetic control during latency and tumorigenesis. We here review what is presently known about the role of epigenetic regulation of viral and host chromatin in KSHV infection and discuss how viral manipulation of these processes may contribute to the development of KSHV-associated disease.

## Introduction

According to current estimates, at least 10% of all worldwide cancer cases are attributable to viral infections [1]. Kaposi sarcoma-associated herpesvirus (KSHV) is one of the eight causative viral species that are presently recognized as class 1 or 2A human carcinogens [2, 3]. The virus was discovered in 1994 by Chang and colleagues [4] in biopsies from AIDS patients suffering from Kaposi sarcoma (KS). KS, first described in 1872 by Moritz Kaposi [5], is an angiosarcoma that had been rare until it saw a dramatic increase in incidence in the wake of the 1980s AIDS epidemic. The incidence has since fallen due to the availability of combination antiretroviral therapies (cART) that efficiently control HIV, yet KS continues to be the most common neoplasm in HIV-positive individuals [6] and remains a leading cause of morbidity and mortality in sub-Saharan Africa. KS is a multifocal tumor with lesions of a very heterogeneous cellular composition (see [7] for a recent review on KS). The proliferating tumor cells in KS lesions, commonly called spindle cells because of their elongated shape, are thought to be of endothelial or mesenchymal origin [8–10]. In addition to spindle cells, the lesions typically also contain abundant inflammatory infiltrates as well as slit-like neovascular spaces. So far, no continuously growing cell line has been established from KS tumors. This may suggest that KS spindle cells are not fully transformed and depend on additional paracrine signals for continued growth.

In addition to KS, KSHV is also linked to two B-cell malignancies: primary effusion lymphoma (PEL) and the plasmablastic form of multicentric Castleman disease (MCD) [11, 12]. PEL is a fully neoplastic disease in which 100% of the tumor cells are monoclonally infected with KSHV. Continuously growing cell lines can be readily established from primary PEL material. These tumor-derived cell lines efficiently maintain KSHV infection and serve as an important model for the study of latency and reactivation [13–15]. In contrast to PEL, plasmablastic MCD is a polyclonal tumor in which nearly all lymph nodes also harbor KSHV-infected B cells that secrete high levels of inflammatory cytokines (reviewed in [16]). In addition to the above cancers, KSHV is also associated with two non-neoplastic, systemic inflammatory diseases: KS immune reconstitution syndrome (KS-IRIS) [17, 18] and KSHV-inflammatory cytokine syndrome (KICS) [19, 20]. Like in MCD, cytokine secretion by KSHV-infected B cells is thought to be at the root of the disease.

Numerous molecular, immunological, and epidemiological studies have provided overwhelming evidence that KSHV is causally linked to the etiology of the above diseases. However, KSHV’s precise contribution to tumorigenic processes, especially regarding cellular transformation, remains unclear. In contrast to some transforming and/or tumorigenic viruses that encode potent oncogenes (e.g., the polyomavirus T antigens or the Epstein Barr Virus (EBV) LMP1 protein), no individual KSHV gene product appears to transform primary human cells by itself. Nevertheless, the ability of several viral proteins and non-coding RNAs to inactivate cellular immune or cell cycle check points (reviewed in [21–25]), together with the fact that tumor-derived PEL cell lines depend on viral gene expression for their survival [26–28], suggests that viral gene products play a pivotal role in the pathogenesis of KSHV-associated tumors. It is therefore most likely that the tumor cells may acquire heritable changes that, in combination with continued viral gene expression, result in malignant transformation. Proto-oncogene or tumor suppressor genes such as TP53, KRAS, or MYC that are frequently mutated in cancers are typically unaffected in PEL and KS tumors [13, 29–34]. Hence, it is an intriguing possibility that epigenetic changes contribute to the pathogenesis of KSHV-associated disease. Given that KSHV encodes many gene products which can modulate host cell chromatin, one of its tumor-promoting activities may be to either directly induce epigenetic alterations or to increase the likelihood with which such changes may occur. In addition, epigenetic modifications of viral chromatin have recently been shown to control latent viral transcription, suggesting that growth-promoting gene expression programs may result from a specific epigenetic profile adopted by viral episomes. We here will review the current knowledge regarding chromatin-modifying activities of KSHV gene products and discuss the role of epigenetic alterations in the viral life cycle and their putative contribution to the pathogenesis of KSHV-associated cancers.

## KSHV: The virus

KSHV is a member of the *Gammaherpesvirin*ae and, together with EBV, forms the human branch of this herpesvirus subfamily. Both EBV and KSHV can cause tumors or hyperproliferative diseases, an ability which they share with several other gammaherpesviruses. Gammaherpesviruses establish long-term reservoirs of infection in lymphocytes (although other cell types, like epithelial cells or fibroblasts, can typically also be infected). It is thought that these viruses may promote host cell proliferation as a strategy to expand latently infected lymphocyte pools and gain access to long-lived memory lymphocyte compartments. In line with this, gammaherpesviruses have been found to employ sophisticated mechanism to ensure latent genome maintenance in proliferating cells, a feature which is also essential for the maintenance of non-integrated episomes in tumor cells.

KSHV has a double-stranded DNA genome of approximately 170 kb. Most of the more than 90 genes encoded by KSHV are required for productive replication. During this phase of the viral life cycle, the so-called lytic genes are transcribed in a cascading manner, resulting in massive amplification of viral genomes, genome packaging, release of new virions, and, ultimately, lysis of the host cell. Like all members of the herpesviruses, KSHV is also able to establish latent infections. Latently infected cells do not produce any viral progeny and remain fully viable, thus allowing the virus to evade host immune surveillance mechanisms and persist for virtually indefinite periods of time. During canonical latency, lytic genes are transcriptionally silenced, and the virus expresses only a minimal set of genes that promote viral persistence and host cell survival. Latent gammaherpesvirus genomes generally persist as non-integrated, fully chromatinized episomes in the nucleus of the infected cells. Since such elements lack a kinetochore, one of the most important functions of latency products is to ensure that viral episomes are properly segregated to daughter cells upon cell division. Indeed, many (if not all) gammaherpesviruses encode latently expressed nuclear proteins that tether viral episomes to mitotic host chromosomes, which thus become piggyback vectors for efficient episome partitioning. Additionally, some of these factors have been demonstrated to directly recruit the cellular replication machinery to viral episomes. In KSHV, these functions are mediated by the multifunctional latency-associated nuclear antigen or LANA. LANA binds via a carboxyterminal domain to distinct binding motifs in the viral terminal repeats (TRs), whereas an amino terminal region interacts with histones H1, H2A, and H2B as well as several other chromatin factors [35–45]. As discussed later, LANA and other latency products can also affect local chromatin states to control cellular gene expression.

In addition to ensuring episome maintenance, KSHV latency products also can promote proliferation and survival of infected host cells. For example, LANA has been reported to interfere with p53, Rb, and β-catenin pathways [46–48]. During the normal viral life cycle, these functions are thought to support expansion of latently infected lymphocyte pools, but they are also likely to play an important role during tumorigenesis. Besides LANA, cells latently infected with KSHV constitutively produce a viral cyclin D homolog (vCyclin), a flice inhibitory-protein-like protein (vFlip), the Kaposin family of proteins, and several highly expressed microRNAs (miRNAs). Like its cellular counterpart, vCyclin can mediate cell cycle progression but is less sensitive to p27, p21, and p16 inhibition [49–52]. vFlip mediates potent antiapoptotic functions via constitutive upregulation of the transcription factor NF-κB [53–56], whereas Kaposins can stabilize AU-rich host transcripts such as cytokine-encoding mRNAs. Finally, the viral miRNAs [57–60] have been shown to antagonize expression of multiple pro-apoptotic, growth inhibitory, or antiviral factors (reviewed in [61–63]). Like their cellular counterparts, they are also able to alter the differentiation status of latently infected cells [64–67].

All of the above genes are produced from alternatively spliced transcripts that originate from a single promoter upstream of the LANA coding region [57–60, 68, 69], a promoter that is constitutively active in KSHV-positive tumor cells as well as in all in vitro latency models studied so far. Besides this core set of canonical latency genes, there are also some genes that are latently expressed in a cell type-specific manner or that are only expressed at low level or in a subfraction of a latently infected population.

These include vIRF3 (also termed LANA2), a protein with homology to human interferon-regulatory factors (IRFs) that is expressed in latently infected B cells, and vIL-6, a viral homolog of the pro-inflammatory cytokine interleukin 6 (IL-6) which is highly expressed in MCD [26, 70–72]. vIRF3 interacts with several cellular IRFs and other transcription factors to antagonize interferon pathways, promote proliferation, and inhibit apoptosis (reviewed in [73]), whereas vIL-6 can signal through the cellular IL-6 receptor even in the absence of its gp80 subunit to exert antiapoptotic and pro-inflammatory functions in a paracrine manner [74].

Given the fact that most proliferating cells in gammaherpesvirus-associated cancers are latently infected, the viral latency program is generally considered the driving force of tumorigenesis. However, despite the many potentially proto-oncogenic functions described above, KSHV latency products alone are unable to robustly transform cells in vitro and also do not efficiently induce tumors in in vivo mouse models. One possible explanation for this fact is that KSHV-encoded latency genes may act in concert with lytic genes to induce tumors. This may seem counterintuitive at first, considering that cells undergoing lytic replication are bound to die. However, several KSHV-encoded factors are secreted from lytic cells or can induce secretion of cellular signaling molecules that can affect other latently infected (or uninfected) cells in a paracrine manner. An important factor among these is vGPCR, a constitutively active G–protein–coupled chemokine receptor homolog [75] which triggers PI3K and p38 MAPK pathways to promote proliferation and angiogenesis (reviewed in [76, 77]). In addition to such paracrine signals, there is mounting evidence that the viral latency program may not be as rigid as previously thought [78–82]. Indeed, depending on the cellular background or the microenvironment, several lytic genes can be expressed outside of the replicative/productive cycle. For example, in vitro infected primary lymphatic endothelial cell (LECs) can adopt a gene expression profile that does not clearly correspond to either canonical latent or lytic transcription programs [82]. Hence, persistently infected cells may be able to adopt alternative forms of viral latency, and conditional expression of alternative latency factors may be an important contributor to transformation and tumorigenesis.

## Epigenetic control in KSHV infection and pathogenesis

The term “epigenetic” describes heritable phenotypic traits or changes that do not result from alterations of the DNA sequence itself. On the molecular level, most epigenetic phenomena are mediated via regulation of gene accessibility and activity through methylation of DNA (which in mammals is usually repressive and almost exclusively occurs at C-G dinucleotides and therefore is also referred to as CpG methylation) or by post-translational histone modifications (histone PTMs) (see [83–88] for reviews). DNA methylation patterns can be autonomously copied beyond replication forks. Due to their mitotic inheritability, such patterns are thus epigenetic in the stricter sense. Importantly, this is not generally true for histone modifications. Although changes in histone PTMs are often universally referred to as epigenetic, many histone PTMs (including histone acetylation) only have a short half-life and are not autonomously transmitted to daughter cells upon cell division. Hence, maintaining such patterns usually requires a sustained trigger, such as continuous binding of a transcription factor. The precise contingent of inheritable histone modifications is not yet known, but it is widely agreed that at least two marks can be considered epigenetic: methylation of histone H3 at lysine 9 or lysine 27 (H3K9 and H3K27, respectively) [89, 90]. In their tri-methylated state, both marks are repressive, but while H3K9-me3 is mainly found in constitutive heterochromatin, H3K27-me3 is a more dynamic facultative heterochromatin mark that is associated with transcriptional repression by polycomb repressive complexes (PRCs) (reviewed in [91–93]).

**Fig. 1:**
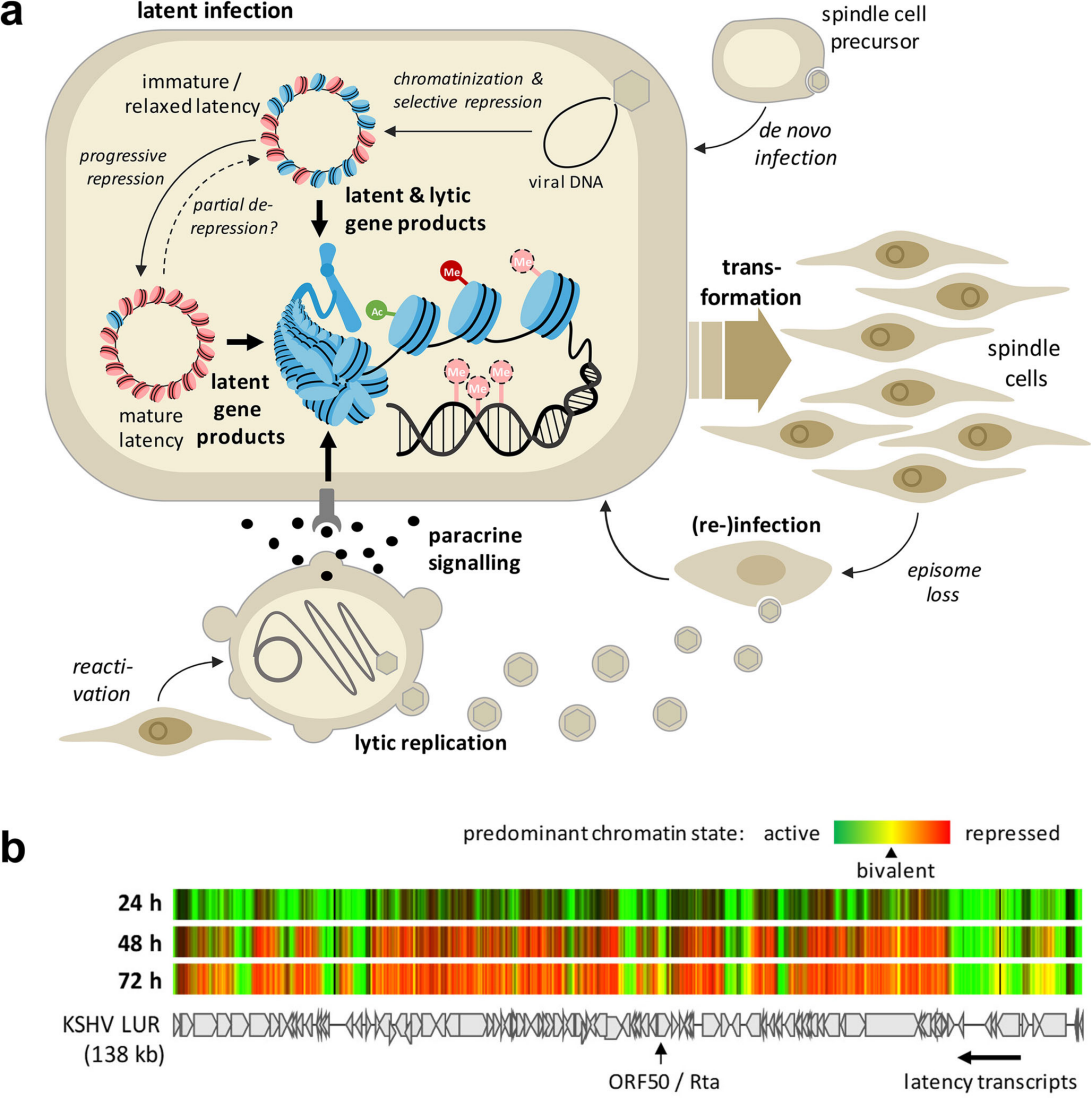
Epigenetic control in KS pathogenesis and latency establishment. **a** Model of epigenetic control mechanisms in KS pathogenesis. Following infection of a spindle cell precursor, KSHV establishes a latent infection. In addition to constitutively expressed latent genes, transient expression of lytic genes during the establishment phase or lytic gene expression that may result from partial de-repression of the viral genome may contribute to epigenetic alteration of host chromatin. Additionally, paracrine signals (e.g., via vIL-6 secretion) received from lytically infected cells may contribute to epigenome alterations. Over time, accumulation of such changes may contribute to tumorigenesis. Additionally, these changes may ensure survival of spindle cells that have lost KSHV until they become re-infected by reactivated cells. **b** Evolution of the viral epigenetic landscape after de novo KSHV infection (adopted from [94], with permission). The heatmap shows accumulation of activating and repressive (H3K4-me3 and H3K27-me3, respectively) histone marks along the KSHV genome at 24-, 48-, and 72-h post-infection. Arrows underneath the map of the KSHV long unique region (LUR) symbolize transcripts encoding constitutive latency genes or the lytic transactivator ORF50/Rta. Activating histone marks (green) are established early in infection, while repressive marks (red) evolve gradually over the 72-h period. Regions marked yellow, e.g., at the ORF50 promoter, carry the hallmarks of bivalent chromatin

When considering the pathogenesis of KSHV-associated tumors (see Fig. 1a for a model of KS), epigenetic control is of interest in the context of viral as well as cellular gene regulation: Firstly, epigenetic mechanisms have been shown to control the viral latency program [94–98]. How the latent viral epigenome itself is established and regulated and to what extend alterations in its profile are directly responsible for the adoption of alternate latency programs are areas of active research. Secondly, several KSHV gene products have been shown to directly or indirectly alter the cellular epigenome. This not only includes canonical latency genes such as LANA but also normally lytic genes that may be stably or transiently expressed under certain conditions during latency. As depicted in Fig. 1a, over time the accumulation of epigenetic alterations in latently infected cells may contribute to the pathogenesis of KSHV-associated tumors. Another potential role relates to the fact that early KS lesions often contain KSHV-positive as well as KSHV-negative spindle cells. It is thought that the latter represent once-infected cells which have lost the virus [99]. If so, it is an attractive hypothesis that virus-induced epigenetic alterations may continue to support proliferation and survival of spindle cells even after the loss of KSHV, at least until they become re-infected by virus particles that are produced by other cells in the lesion.

## Epigenetic regulation of latent gene expression

**Fig. 2:**
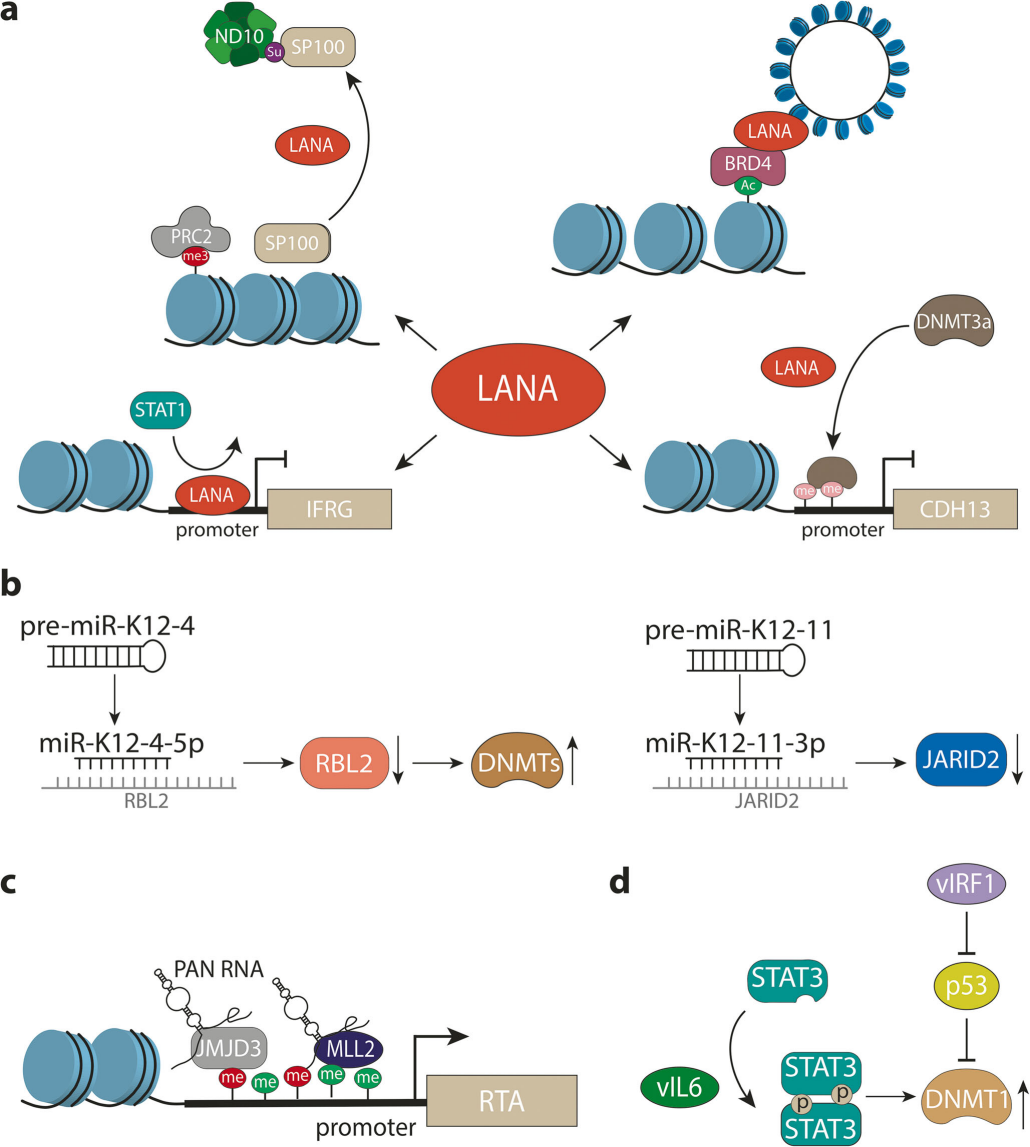
Examples of interactions between viral proteins and cellular epigenetic pathways. **a** Selected epigenetic or chromatin-associated functions of LANA (see text for further details). *Top right:* LANA interacts with BRD4 and other BET family members, presumably to tether viral episomes to euchromatic regions. *Lower right:* LANA leads to hypermethylation of the CDH13 promoter, likely via recruitment of Dnmt3A. *Lower left:* LANA binds to the promoter of interferon-regulated genes (IFRG) and prevents activation, presumably by interfering with Stat1 binding. *Upper left:* LANA induces sumoylation of Sp100, resulting in relocalization of chromatin-bound Sp100 into the insoluble matrix (likely to ND10 bodies) and accelerated accumulation of H3K27-me3 marks on viral genomes. **b***Left*: miR-K12-4-5p inhibits expression of Rbl2, a repressor of Dnmt expression. *Right:* miR-K12-11-3p represses expression of Jarid2, a conditional component of PRC2 complexes. **c** The viral lncRNA Pan recruits the H3K27-specific demethylases JMJD3 and UTX (not shown) as well as the H3K4 methyltransferase MLL2 to activate promoter of the gene encoding Rta (ORF50). **d** vIL-6 and vIRF3 upregulate Dnmt1 expression via Stat3 activation or p53 inhibition, respectively

As for all herpesviruses, the viral DNA found in KSHV virions is not wrapped in histones and completely free of CpG methylation. Hence, genomes delivered to the nucleus of a newly infected cell are epigenetically naïve, and latent chromatin must be re-established upon each round of infection. Based on the observation that discrete loci of KSHV genomes in PEL cell lines are methylated, it had originally been thought that DNA methylation might be responsible for the silencing of lytic genes during latency establishment. However, using genome-wide analyses with methylated DNA and chromatin immunoprecipitation (MeDIP and ChIP, respectively), we were able to demonstrate that appreciable CpG methylation patterns do not emerge until several weeks following a de novo infection. Instead, viral episomes rapidly and globally acquire H3K27-me3 marks early in infection [95]. The enrichment of H3K27-me3 is highly significant, and overall patterns are very similar when compared between PEL cells and various in vitro infected cell lines or primary cells [94–98]. Only a few loci (including the major latency promoter upstream of the LANA-coding region) escape PRC-mediated silencing and instead maintain distinct peaks of activating histone marks such as H3K4-me3 [95, 96]. Interestingly, whereas the activating marks are established within a few hours of infection, repressive H3K27-me3 marks gradually evolve during the first 48 to 72 h (see [94, 97] and heat map in Fig. 2b). This observation suggests that early viral chromatin undergoes stepwise maturation before the fully restricted latency state is established. In line with this, previous studies have shown that, during the first hours of a de novo infection, KSHV exhibits a relaxed expression profile, which includes many lytic genes [100]. Therefore, it is possible that combined activities of latent and lytic gene products during early infection may permanently affect the host cell via alteration of the host epigenome.

An important feature of H3K27-me3-positive facultative heterochromatin is that it is more dynamic when compared to constitutive heterochromatin. Studies in various organisms have shown that repression by polycomb complexes can be overcome relative easily, and some PRC-bound genes in mammals have been found to frequently switch between silent and transcriptional active states [101]. These observations indicate that polycomb repression may primarily serve to dampen transcription, rather than switching genes completely off. The obvious benefit of adopting such a state during KSHV latency is that lytic genes can be rapidly re-expressed once conditions in the host cell become unfavorable. Indeed, the promoter of the viral master switch lytic transactivator Rta simultaneously maintains activating H3K4-me3 as well as repressive H3K27-me3 marks [95, 96]. Such bivalent chromatin states are typically found on differentiation-associated genes in embryonic stem cells and are known to be poised for rapid reactivation [102].

Given the above, it is likely that KSHV latency represents a rather flexible instead of rigid transcriptional program. This is in line with the observation that the signaling molecules K1 and K15, genes which are highly expressed during the lytic cycle, can also be transcribed at low level in latently infected cell populations [78–81, 103, 104]. Whether or not these transcription signatures stem from weak but uniform transcription in all cells or from transient switching of promoters to a fully active state in a minority of the cells is not yet known. Likewise, it is presently unknown to what extend alternative latency modes may depend upon an altered epigenetic profile of viral chromatin. Global anticorrelation of H3K4-me3 and H3K27-me3 marks (Fig. 2b) and the observation that establishment of activating marks precedes that of H3K27-me3 [94, 96, 97] suggest that the early binding of (as of yet unknown) transcription factors is able to create regions of constitutively open chromatin that are protected from polycomb repression. An altered or cell-type–specific spectrum of transcription factors would therefore be expected to result in altered epigenetic profiles. Even in the absence of constitutive changes, the overall plasticity of polycomb-repressed chromatin indicates that latent KSHV genomes may be able to fluctuate between fully silenced and relaxed states, potentially allowing for stochastic firing of lytic promoters. If so, similar to the early phase of infection, there may be intermittent phases when both latent and lytic genes are co-expressed and act upon host cell chromatin.

How are PRCs attracted to KSHV episomes and to what extend is this process controlled by the virus? Our own recent data suggest that the composition of the viral genome sequence itself favors rapid silencing by PRCs [98]. There are two major forms of polycomb repressive complexes, PRC1 and PRC2. PRC2 catalyzes tri-methylation of H3K27 via its EZH2 component, while PRC1 can bind to resulting H3K27-me3 marks and cooperate with PRC2 to mediate repression. In the canonical recruitment pathway, primary targeting is therefore mediated by PRC2. However, whereas discrete PRC2 recruitment elements exist in insect cells, similar signals in mammalian genomes have long remained elusive (reviewed in [105]). Instead, it is now becoming increasingly clear that high density of unmethylated CpG motifs, in particular in CpG islands, represents a major mammalian factor for direct recruitment of PRC1 as well as PRC2 [105–114]. This is of interest since KSHV genomes are very CpG rich molecules that enter the nucleus in a completely unmethylated state. Indeed, the entire KSHV genome effectively resembles one contiguous CpG island [98]. In accordance with this, we could recently demonstrate that KDM2B, a PRC component that directly binds to non-methylated CpGs [110, 111, 113], associates with KSHV genomes very early during infection. In contrast, a related herpesvirus with a substantially lower CpG density did not show evidence of KDM2B binding and consequently did also not acquire repressive H3K27-me3 marks [98]. In addition to these cis-acting sequence features, there is also evidence that latent KSHV gene products favor PRC recruitment. For example, LANA has been found to directly bind to PRC2 complexes, which may potentially favor polycomb repression via increasing the local concentration of PRC around viral genomes [115]. While this activity requires binding to viral chromatin, other LANA functions such as upregulation of EZH2 or re-localization of Sp100 (discussed in the next section) may be more pleiotropic and therefore may ultimately also affect the cellular epigenome [94].

## Viral manipulation of the host epigenome

The investigation of KSHV-induced cellular epigenome changes, especially in relevant primary cell types, is still a relatively young research field. In large part, this is due to the limited availability of suitable infection systems. Primary lymphocytes are largely refractory to KSHV infection in vitro, a fact that greatly complicates the study of potential B-cell-specific mechanisms. In contrast, primary endothelial cells or mesenchymal stem cells (thought to be potential precursors of KS spindle cells) can be infected in vitro, yet long-term investigation of such cultures is difficult because they are not efficiently immortalized by KSHV and furthermore tends to lose the virus after only a few cell doublings. Most studies have therefore used de novo infection or ectopic expression of viral genes in fully transformed cell lines instead. Hence, a potential caveat is that some of the observations made in these systems could represent artifacts resulting from the use of heterologous cell lines or ectopic overexpression. We will discuss some of the activities which may contribute to the pathogenesis of KSHV-associated disease in the following. In Table 1, we furthermore present an overview of known interactions between KSHV gene products and epigenetic or chromatin regulatory pathways, together with the model systems in which they were observed.

**Table 1:** Interplay between latent and lytic KSHV gene products and host epigenetic pathways

Viral Factor	Reported Function(s)	Model system(s)
*LANA* *(latent)*	LANA recruits NAP1L1 to viral terminal repeats to regulate nucleosome assembly and gene expression [116]	PEL cell lines (BCBL-1, BC-3); ectopic expression (HEK293T, HEK293L)
	LANA interferes with the interaction between CIITA and RFX components, resulting in reduced MHC-II promoter activity [117]	PEL cell lines (BCBL-1, BC-3); ectopic expression (HEK293T, HEK293L, BJAB, THP-1, and DG75)
	LANA interacts with the H3K4 methyltransferase complex hSET1, binds preferentially to viral and cellular chromatin at H3K4me3-positive loci [118]	PEL cell lines (BCBL-1, BC-3); in vitro infection (TIVE); ectopic expression (HEK293, BJAB)
	LANA localizes to heterochromatic regions within the nucleus [119]	PEL cell lines (BCBL-1, BC-1); in vitro infection (ECV, K562)
	LANA downregulates viral lytic genes, recruits PRC2 to viral episomes during de novo infection [115]	In vitro infection (SLK, iSLK, TIME); ectopic expression (HEK293T)
	LANA facilitates PRC2-recruitment to viral episomes via relocalization of Sp100 into insoluble matrix fractions [94]	PEL cell lines (BCBL-1, HBL6, Cro-AP/2); in vitro infection (SLK, HDF, HUVEC, EA.hy 926); in vitro transfection with bacmid DNA (SLK); ectopic expression (EA.hy 926, HeLa)
	LANA downregulates TGF-ß signaling by increasing DNA methylation at the TßRII promoter [120]	PEL cell lines (BCBL-1, BC-1, BC-2, BC-3, BC-5); in vitro infection (TIVE); ectopic expression (BJAB)
	LANA recruits DNMT3a and increases DNA methylation at cellular promoters (e.g., CCND2 and CDH13) [121]	PEL cell lines (BCBL-1, BC-3, JSC-1); ectopic expression (TIME, HEK293T)
	LANA interacts with the H3K9me1/2 demethylase KDM3A [122]	PEL cell line (BCBL-1); ectopic expression (HeLa, HEK293T); in vitro infection (Vero, HEK293T)
	LANA interacts with the H3K9 methyltransferase SUV39H1 at the terminal repeats [123]	PEL cell line (BC-3); ectopic expression (HEK293, Vero)
	LANA interacts with HP-1 [123, 124]	Ectopic expression (HEK293T, C33A)
	LANA recruits KAP1 to the viral genome, resulting in decreased gene expression [125]	PEL cell lines (BCBL-1, JSC-1, BC-3); in vitro infection (HeLa); ectopic expression (HEK293T, HeLa)
	LANA alters the higher organization of host cell chromatin [126]	PEL cell lines (BCBL-1, BC-1); ectopic expression (MCF7, HeLa, Saos-2, A9, L, NIH3T3)
	LANA interacts with members of the BET protein family (BRD2, BRD3, and BRD4) and can release BRD4-mediated cell cycle arrest [127]	PEL cell lines (BCBL-1, JSC-1, BCP-1, CroAP-5); ectopic expression (HEK293T, BJAB)
	LANA association with BRD2 and BRD4 is critical for viral latency, treatment with BET-inhibitors results in lytic reactivation [128]	PEL cell lines (BCBL-1, BC-1, BC-3, JSC-1); in vitro infection (BJAB, SLK)
	LANA prevents Bub1-mediated phosphorylation of H2A at position T120 to influence Sgo1 localization, resulting in chromosomal instability [129]	PEL cell lines (BC-3, JSC-1); in vitro infection (BJAB, HT1080); ectopic expression (BJAB, HEK293, HT1080)
*LANA & vFLIP* *(latent)*	LANA and vFlip cooperatively upregulate EZH2 in a NF-kB dependent manner to induce angiogenesis [130]	In vitro infection (SLK, BOEC); ectopic expression (BOEC)
*miRNAs* *(latent)*	miR-K12–11 targets JARID2 [67]	PEL cell line (BCBL-1); ectopic expression in vitro (NIH 3 T3, HEK293T); transgenic expression in vivo (C57BL/6 mice)
	miR-K12–4-5p targets RBL2, thereby increasing DNMT levels [131]	In vitro transfection/infection (HEK293); ectopic expression (HEK293)
*vIRF3* *(latent/ lytic)*	vIRF3 reduces HDAC5 phosphorylation which plays a role in viral induced angiogenesis [132]	In vitro (Bac16) infection (LECs); ectopic expression (LECs, BECs, TREx-BCBL-1, HeLa)
*vIL6* *(latent/ lytic)*	vIL6 upregulates the DNA methyltransferase DNMT1, resulting in an increase of global DNA methylation [133]	Ectopic expression (EA.hy926)
*vIRF1* *(lytic)*	vIRF1 upregulates DNMT1 via downregulation of p53 to increase DNA methylation in the miR-218 promoter [134]	In vitro (KSHVwt and Bac16) infection (HUVEC); ectopic expression (HUVEC)
*PAN RNA* *(lytic)*	PAN RNA interacts with several histone modifying enzymes (MLL2, UTX, and JMJD3) and can transcriptionally activate viral genes (e.g., RTA) [135]	PEL cell lines (TREx/BCBL-1 RTA); in vitro transfection (HEK293L); ectopic expression (HEK293L)
	PAN RNA regulates several host pathways (e.g., cell cycle) by binding to host as well as to viral chromatin and interacts with members of the PRC2 complex [136]	PEL cell lines (TREx/BCBL-1 RTA); in vitro infection (PBMC); in vitro transfection with bacmid DNA (HEK293); ectopic expression (BJAB, Jurkat, THP1, RPE)
	PAN RNA interacts with several host proteins like histones H1 and H2A [137]	PEL cell lines (BCBL-1); ectopic expression (HEK293, BJAB)
*RTA* *(lytic)*	RTA binds to GMNN, a protein involved in cell cycle and chromatin remodeling [138]	PEL cell lines (TRExBCBL1-3xFLAG-RTA); in vitro infection (iSLK); ectopic expression (HEK293T, BJAB, iSLK)

Given its constitutive expression and close association with chromatin, LANA is one of the factors which are most likely to influence the cellular epigenome (see Fig. 2a for a graphical illustration of selected LANA-associated functions). Indeed, LANA has been found to positively or negatively influence expression of many human genes, and in some cases, this regulation has been linked to changes in histone modification or DNA methylation patterns. For example, LANA has been reported to inhibit TGF-β signaling by inducing DNA methylation at Sp-1 binding sites within the promoter of the TGF-β type II receptor (TβRII) [120]. Methylation had first been detected in PEL cell lines and was subsequently demonstrated in LANA-expressing telomerase-immortalized umbilical-vein endothelial (TIVE) cells. The observation that treatment with demethylating agents sensitizes PEL lines to apoptosis and that primary cases of PEL, KS, and MCD also exhibited decreased levels of TβRII strongly suggests that epigenetic silencing of the TβRII promoter contributes to the pathogenesis of KSHV-associated tumors [120]. In another study, constitutive LANA expression in telomerase-immortalized microvascular endothelial (TIME) cells was found to lead to methylation and repression of the promoter of CDH13, the gene encoding H-cadherin (a protein with growth inhibitory functions) [121]. The promoter was also found to be methylated in PEL lines, and since LANA was furthermore demonstrated to interact with the de novo DNA methyltransferase (Dnmt) 3a, it was suggested that LANA may mediate repression by directly targeting Dnmt3a to the CDH13 (and other) cellular promoters. It seems likely that such a mechanism is also responsible for TβRII silencing; however, this has not been formally demonstrated so far.

Several other studies have employed ChIP-seq techniques to investigate the interaction of LANA with host cell chromatin in endothelial cells and PEL cell lines on a more global level [118, 139, 140]. Although there is not necessarily a large overlap between the identified sites, all studies agree on several points: Firstly, LANA was found to bind a significant number (hundreds to thousands) of host sites. Secondly, de novo infection experiments suggest that LANA predominantly binds to sites that are already in an open chromatin formation prior to infection, an observation which may reflect the interaction of LANA with BET proteins [44, 127, 141] or the H3K4 methyltransferase hSET1 [118]. It is therefore possible that the tethering function of LANA may preferentially target viral episomes to euchromatic host loci, perhaps to prevent abrogation of latent gene expression. Thirdly, most genes that were located close to LANA binding sites did not exhibit significant transcriptional changes. This observation suggests that silencing of gene expression via recruitment of DNA methyltransferases is not the universal outcome of LANA binding but instead may be restricted to a few host genes such as TβRII and CDH13 [120, 121], perhaps in a context-specific manner (of note, these two sites were not recovered in the three ChIP-seq studies, but this may be due to the higher sensitivity of the specific PCR that was used in the previous studies). While LANA binding therefore seems to be indolent at most sites, it is interesting that one study observed LANA-binding peaks which partially overlapped with Stat1-binding sites in the promoters of gamma interferon (IFNγ) regulated genes. Indeed, IFNγ treatment demonstrated that LANA could counteract activation of a subset of these genes, suggesting that some of the chromatin changes associated with KSHV infection may conditionally alter gene expression only after activation of specific pathways [140].

In addition to site-specific effects, LANA may also affect host chromatin in a more indirect manner. For example, LANA was found to efficiently release Sp100, a component of ND10 nuclear bodies, from the soluble chromatin fraction and re-localize it into the insoluble matrix [94]. The fact that this phenotype can be observed in de novo infected cells as well as PEL cell lines suggests that permanent re-localization of Sp100 is a general feature of KSHV latency. Since Sp100 depletion results in accelerated acquisition of H3K27-me3 by viral episomes, the re-localization likely serves to favor latency establishment. Whether or not the permanent removal of Sp100 from soluble chromatin fractions also has epigenetic consequences for the host genome has not been explored so far. LANA has also been shown to cooperate with vFlip in the transcriptional upregulation of EZH2, the H3K27-specific methyltransferase of PRC2 complexes [130]. In accordance with this finding, the protein was demonstrated to be highly expressed in KS tissues, and upregulation of EZH2 by KSHV was found to be required for the induction of tube formation in infection blood outgrowth endothelial cells (BOECs). Although H3K27-me3 levels in BOECs were generally increased, however, it is presently unknown whether silencing of specific target genes by PRC2 is responsible for the phenotype.

Lastly, a recent publication found that LANA interacts with Bub1, a component of the spindle checkpoint complex, to inhibit phosphorylation of histone 2A at residue T120 (H2AT120) [129]. LANA-dependent inhibition of H2AT120 phosphorylation resulted in dislocation of Sgo1, a protein which protects centromere integrity, and induction of aneuploidy. Thus, LANA may promote tumorigenesis by inducing epigenetic alterations as well as chromosomal instability.

Besides LANA and vFlip, KSHV-encoded miRNAs have also been found to affect the cellular epigenome (Fig. 2b). Strikingly, infection with a mutant virus unable to express the miRNAs resulted in an almost complete loss of DNA methylation within the viral as well as the cellular genome [131]. A potential explanation for this observation is that expression of one of the KSHV miRs (miR-K12-4-5p) can inhibit expression of Rbl2, a known repressor of Dnmts, thereby leading to upregulation of Dnmt1, − 3a and – 3b. Another KSHV miRNA, miR-K12-11, represents a viral mimic of the human miR-155, an evolutionary conserved proto-oncogenic miRNA which is overexpressed in many lymphocyte malignancies [64, 65, 67]. Among the targets shared by miR-K12-11 and miR-155 is Jarid2, a conditional component of PRC2 [67]. Interestingly, Jarid2 inhibition by miR-155 has been found to activate cytokine expression in TH17 cells [142]. The epigenetic consequences of miR-K12-11-induced repression of Jarid2 in KSHV-infected B cells, however, have not yet been investigated.

In addition to the above latency genes, several lytic gene products can affect viral and host chromatin (see Table 1 and Fig. 2b–d). For example, the nuclear PAN RNA is a long non-coding RNA (lncRNA) which is very strongly expressed in lytic cells, but that can also be detected at lower levels during latency [136]. PAN has been found to bind to several viral and host loci and to not only interact with the PRC2 components EZH2 and Suz12 but also with the H3K27-specific demethylases UTX and JMJD3 and the H3K4-me3 methyltransferase MLL2 [135–137]. Thus, PAN may promote as well as counteract PRC repression, a hypothesis which is in line with the observation that Pan supports lytic KSHV gene expression while also repressing many immune regulatory genes. Hence, like other lncRNAs, PAN may act as a molecular scaffold to mediate epigenetic changes to up- or downregulate gene expression in a context-dependent manner. The mechanisms mediating specific targeting of PAN and associated epigenetic modifiers to promoters, however, remain unknown. Two other studies showed that vIL-6 and vIRF1, two predominantly lytic factors that are also weakly expressed during latency, can mediate upregulation of Dnmt1 (the DNA methyltransferase responsible for maintaining methylation patterns during cell division) to promote cell proliferation, migration and invasiveness [133, 134]. For vIL-6, upregulation is dependent upon Stat3 activation, whereas vIRF1 induced Dnmt1 via p53 inhibition. In accordance with increased Dnmt1 expression, vIRF1 expression was also shown to result in hypermethylation of the pre-miR-218-1 promoter, which in turn leads to increased expression of the miR-218-5p targets high mobility group box 2 (HMGB2) and cytidine/uridine monophosphate kinase 1 (CMPK1), two proteins that are upregulated in various tumors [134].

Of note, a study of global DNA methylation patterns in BJAB cells (an EBV-negative Burkitt’s lymphoma cell line) that had been infected with a recombinant KSHV also observed transcriptional changes that correlated with methylation changes in the promoters of several genes [121]. Approximately 70% of a total of 452 differentially expressed genes were transcriptionally repressed and hypermethylated, indicating that de novo KSHV may favor increased DNA methylation overall.

Although the above findings very strongly suggest that epigenome alterations have a profound effect on viral gene expression and infection-associated tumorigenesis, all hitherto discussed observations have been made in in vitro systems. What is the evidence that such changes also matter in vivo? An interesting study by Naipauer and colleagues [143] has demonstrated that KSHV-infected mesenchymal stem cells can form tumors in nude mice but only if they are grown in KS-like (i.e., cytokine-rich) medium prior to injection. Indeed, these cells adopt a relaxed expression profile that includes expression of many lytic genes, apparently accompanied by global decrease of H3K27-me3 on viral genomes. At the same time, KSHV-infected cells exhibit changes of H3K27-me3 levels in the promoters of many host genes involved in pathways relevant to KS pathogenesis (e.g., VEGF, p53, Toll-like receptor of IFN signaling). While many questions remain, this study suggests that mesenchymal stem cells represent a very attractive in vivo model to not only study the influence of environmental clues on epigenetic control of viral gene expression but also host epigenome alterations that may be caused by expression of an extended repertoire of viral genes.

## Conclusion

There is profound evidence that control of latent KSHV infection is intricately linked to epigenetic regulation. Although some of the mechanisms which shape the epigenetic landscape of viral genomes have been discovered, important questions regarding viral epigenome dynamics and adoption of alternate, potentially tumor-promoting latency programs remain. This is particularly true because constitutive latency genes may cooperate with transiently expressed lytic factors to induce stable and heritable changes of the cellular epigenome. Studying the combinatorial effects of such genes, especially in relevant primary cell models, represents a promising direction for future research. In addition to existing in vitro models of endothelial cell infection, newly developed mesenchymal stem cell systems may provide novel opportunities for in vivo studies. Since the majority of PELs also harbors EBV, another interesting yet unexplored aspect is the effect of combined KSHV and EBV gene expression on viral and host epigenomes that can be studied in recently established B-cell models of co-infection [144]. The availability of these systems, together with recent technology advances that allow epigenomic and transcriptomic analysis at single cell resolution, represents new and exciting possibilities to study the role of epigenetics in infection and the pathogenesis of virus-associated cancers.

## References

[1] Plummer M, de Martel C, Vignat J, Ferlay J, Bray F, Franceschi S (2016). Global burden of cancers attributable to infections in 2012: a synthetic analysis. Lancet Glob Health.

[2] Iarc working group on the evaluation of carcinogenic risks to humans (2012) biological agents. Volume 100 B. A review of human carcinogens. IARC Monogr Eval Carcinog Risks Hum 100(Pt B):1–441 PMC4781184 23189750

[3] Iarc Working Group on the Evaluation of Carcinogenic Risks to Humans (2014). Malaria and some Polyomaviruses (Sv40, Bk, Jc, and Merkel cell viruses). IARC Monogr Eval Carcinog Risks Hum.

[4] Chang Y, Cesarman E, Pessin MS, Lee F, Culpepper J, Knowles DM, Moore PS (1994). Identification of herpesvirus-like DNA sequences in AIDS-associated Kaposi's sarcoma. Science.

[5] Kaposi M (1872). Idiopathisches multiples Pigmentsarkom der haut. Arch f Dermat.

[6] Silverberg MJ, Lau B, Achenbach CJ, Jing Y, Althoff KN, D'Souza G, Engels EA, Hessol NA, Brooks JT, Burchell AN, Gill MJ, Goedert JJ, Hogg R, Horberg MA, Kirk GD, Kitahata MM, Korthuis PT, Mathews WC, Mayor A, Modur SP, Napravnik S, Novak RM, Patel P, Rachlis AR, Sterling TR, Willig JH, Justice AC, Moore RD, Dubrow R, North American ACCoR, Design of the International Epidemiologic Databases to Evaluate A (2015). Cumulative incidence of cancer among persons with HIV in North America: a cohort study. Ann Intern Med.

[7] Cesarman E, Damania B, Krown SE, Martin J, Bower M, Whitby D (2019). Kaposi sarcoma. Nat Rev Dis Primers.

[8] Kahn HJ, Bailey D, Marks A (2002). Monoclonal antibody D2-40, a new marker of lymphatic endothelium, reacts with Kaposi's sarcoma and a subset of angiosarcomas. Mod Pathol.

[9] Pyakurel P, Pak F, Mwakigonja AR, Kaaya E, Heiden T, Biberfeld P (2006). Lymphatic and vascular origin of Kaposi's sarcoma spindle cells during tumor development. Int J Cancer.

[10] Li Y, Zhong C, Liu D, Yu W, Chen W, Wang Y, Shi S, Yuan Y (2018). Evidence for Kaposi sarcoma originating from mesenchymal stem cell through KSHV-induced mesenchymal-to-endothelial transition. Cancer Res.

[11] Cesarman E, Chang Y, Moore PS, Said JW, Knowles DM (1995). Kaposi's sarcoma-associated herpesvirus-like DNA sequences in AIDS-related body-cavity-based lymphomas. N Engl J Med.

[12] Soulier J, Grollet L, Oksenhendler E, Cacoub P, Cazals-Hatem D, Babinet P, d'Agay MF, Clauvel JP, Raphael M, Degos L (1995). Kaposi's sarcoma-associated herpesvirus-like DNA sequences in multicentric Castleman's disease. Blood.

[13] Cesarman E, Moore PS, Rao PH, Inghirami G, Knowles DM, Chang Y (1995). In vitro establishment and characterization of two acquired immunodeficiency syndrome-related lymphoma cell lines (BC-1 and BC-2) containing Kaposi's sarcoma-associated herpesvirus-like (KSHV) DNA sequences. Blood.

[14] Arvanitakis L, Mesri EA, Nador RG, Said JW, Asch AS, Knowles DM, Cesarman E (1996). Establishment and characterization of a primary effusion (body cavity-based) lymphoma cell line (BC-3) harboring kaposi's sarcoma-associated herpesvirus (KSHV/HHV-8) in the absence of Epstein-Barr virus. Blood.

[15] Renne R, Zhong W, Herndier B, McGrath M, Abbey N, Kedes D, Ganem D (1996). Lytic growth of Kaposi's sarcoma-associated herpesvirus (human herpesvirus 8) in culture. Nat Med.

[16] Uldrick TS, Polizzotto MN, Yarchoan R (2012). Recent advances in Kaposi sarcoma herpesvirus-associated multicentric Castleman disease. Curr Opin Oncol.

[17] Bower M, Nelson M, Young AM, Thirlwell C, Newsom-Davis T, Mandalia S, Dhillon T, Holmes P, Gazzard BG, Stebbing J (2005). Immune reconstitution inflammatory syndrome associated with Kaposi's sarcoma. J Clin Oncol.

[18] Connick E, Kane MA, White IE, Ryder J, Campbell TB (2004). Immune reconstitution inflammatory syndrome associated with Kaposi sarcoma during potent antiretroviral therapy. Clin Infect Dis.

[19] Polizzotto MN, Uldrick TS, Wyvill KM, Aleman K, Marshall V, Wang V, Whitby D, Pittaluga S, Jaffe ES, Millo C, Tosato G, Little RF, Steinberg SM, Sereti I, Yarchoan R (2016). Clinical features and outcomes of patients with symptomatic Kaposi sarcoma herpesvirus (KSHV)-associated inflammation: prospective characterization of KSHV inflammatory cytokine syndrome (KICS). Clin Infect Dis.

[20] Uldrick TS, Wang V, O'Mahony D, Aleman K, Wyvill KM, Marshall V, Steinberg SM, Pittaluga S, Maric I, Whitby D, Tosato G, Little RF, Yarchoan R (2010). An interleukin-6-related systemic inflammatory syndrome in patients co-infected with Kaposi sarcoma-associated herpesvirus and HIV but without multicentric Castleman disease. Clin Infect Dis.

[21] Cesarman E (2014). How do viruses trick B cells into becoming lymphomas?. Curr Opin Hematol.

[22] Schulz TF, Cesarman E (2015). Kaposi sarcoma-associated herpesvirus: mechanisms of oncogenesis. Curr Opin Virol.

[23] Cavallin LE, Goldschmidt-Clermont P, Mesri EA (2014). Molecular and cellular mechanisms of KSHV oncogenesis of Kaposi's sarcoma associated with HIV/AIDS. PLoS Pathog.

[24] Jung J, Munz C (2015). Immune control of oncogenic gamma-herpesviruses. Curr Opin Virol.

[25] Günther T, Grundhoff A (2017) Epigenetic manipulation of host chromatin by Kaposi sarcoma-associated herpesvirus: a tumor-promoting factor? Curr Opin Virol 26:104–111. 10.1016/j.coviro.2017.07.018 10.1016/j.coviro.2017.07.018 28802146

[26] Wies E, Mori Y, Hahn A, Kremmer E, Sturzl M, Fleckenstein B, Neipel F (2008). The viral interferon-regulatory factor-3 is required for the survival of KSHV-infected primary effusion lymphoma cells. Blood.

[27] Guasparri I, Keller SA, Cesarman E (2004). KSHV vFLIP is essential for the survival of infected lymphoma cells. J Exp Med.

[28] Keller SA, Schattner EJ, Cesarman E (2000). Inhibition of NF-kappaB induces apoptosis of KSHV-infected primary effusion lymphoma cells. Blood.

[29] Brimo F, Michel RP, Khetani K, Auger M (2007). Primary effusion lymphoma: a series of 4 cases and review of the literature with emphasis on cytomorphologic and immunocytochemical differential diagnosis. Cancer.

[30] Katano H, Sato Y, Sata T (2001). Expression of p53 and human herpesvirus-8 (HHV-8)-encoded latency-associated nuclear antigen with inhibition of apoptosis in HHV-8-associated malignancies. Cancer.

[31] Petre CE, Sin SH, Dittmer DP (2007). Functional p53 signaling in Kaposi's sarcoma-associated herpesvirus lymphomas: implications for therapy. J Virol.

[32] Li JJ, Huang YQ, Cockerell CJ, Zhang WG, Nicolaides A, Friedman-Kien AE (1997). Expression and mutation of the tumor suppressor gene p53 in AIDS-associated Kaposi's sarcoma. Am J Dermatopathol.

[33] Scinicariello F, Dolan MJ, Nedelcu I, Tyring SK, Hilliard JK (1994). Occurrence of human papillomavirus and p53 gene mutations in Kaposi's sarcoma. Virology.

[34] Nicolaides A, Huang YQ, Li JJ, Zhang WG, Friedman-Kien AE (1994). Gene amplification and multiple mutations of the K-ras oncogene in Kaposi's sarcoma. Anticancer Res.

[35] Hu J, Garber AC, Renne R (2002). The latency-associated nuclear antigen of Kaposi's sarcoma-associated herpesvirus supports latent DNA replication in dividing cells. J Virol.

[36] Grundhoff A, Ganem D (2003). The latency-associated nuclear antigen of Kaposi's sarcoma-associated herpesvirus permits replication of terminal repeat-containing plasmids. J Virol.

[37] Ballestas ME, Chatis PA, Kaye KM (1999). Efficient persistence of extrachromosomal KSHV DNA mediated by latency-associated nuclear antigen. Science.

[38] Barbera AJ, Chodaparambil JV, Kelley-Clarke B, Joukov V, Walter JC, Luger K, Kaye KM (2006). The nucleosomal surface as a docking station for Kaposi's sarcoma herpesvirus LANA. Science.

[39] Lim C, Sohn H, Lee D, Gwack Y, Choe J (2002). Functional dissection of latency-associated nuclear antigen 1 of Kaposi's sarcoma-associated herpesvirus involved in latent DNA replication and transcription of terminal repeats of the viral genome. J Virol.

[40] Stedman W, Deng Z, Lu F, Lieberman PM (2004). ORC, MCM, and histone hyperacetylation at the Kaposi's sarcoma-associated herpesvirus latent replication origin. J Virol.

[41] Cotter MA, Robertson ES (1999). The latency-associated nuclear antigen tethers the Kaposi's sarcoma-associated herpesvirus genome to host chromosomes in body cavity-based lymphoma cells. Virology.

[42] Krithivas A, Fujimuro M, Weidner M, Young DB, Hayward SD (2002). Protein interactions targeting the latency-associated nuclear antigen of Kaposi's sarcoma-associated herpesvirus to cell chromosomes. J Virol.

[43] Xiao B, Verma SC, Cai Q, Kaul R, Lu J, Saha A, Robertson ES (2010). Bub1 and CENP-F can contribute to Kaposi's sarcoma-associated herpesvirus genome persistence by targeting LANA to kinetochores. J Virol.

[44] Viejo-Borbolla A, Ottinger M, Bruning E, Burger A, Konig R, Kati E, Sheldon JA, Schulz TF (2005). Brd2/RING3 interacts with a chromatin-binding domain in the Kaposi's sarcoma-associated herpesvirus latency-associated nuclear antigen 1 (LANA-1) that is required for multiple functions of LANA-1. J Virol.

[45] You J, Srinivasan V, Denis GV, Harrington WJ, Ballestas ME, Kaye KM, Howley PM (2006). Kaposi's sarcoma-associated herpesvirus latency-associated nuclear antigen interacts with bromodomain protein Brd4 on host mitotic chromosomes. J Virol.

[46] Friborg J, Kong W, Hottiger MO, Nabel GJ (1999). p53 inhibition by the LANA protein of KSHV protects against cell death. Nature.

[47] Radkov SA, Kellam P, Boshoff C (2000). The latent nuclear antigen of Kaposi sarcoma-associated herpesvirus targets the retinoblastoma-E2F pathway and with the oncogene Hras transforms primary rat cells. Nat Med.

[48] Fujimuro M, Wu FY, ApRhys C, Kajumbula H, Young DB, Hayward GS, Hayward SD (2003). A novel viral mechanism for dysregulation of beta-catenin in Kaposi's sarcoma-associated herpesvirus latency. Nat Med.

[49] Chang Y, Moore PS, Talbot SJ, Boshoff CH, Zarkowska T, Godden K, Paterson H, Weiss RA, Mittnacht S (1996). Cyclin encoded by KS herpesvirus. Nature.

[50] Godden-Kent D, Talbot SJ, Boshoff C, Chang Y, Moore P, Weiss RA, Mittnacht S (1997). The cyclin encoded by Kaposi's sarcoma-associated herpesvirus stimulates cdk6 to phosphorylate the retinoblastoma protein and histone H1. J Virol.

[51] Li M, Lee H, Yoon DW, Albrecht JC, Fleckenstein B, Neipel F, Jung JU (1997). Kaposi's sarcoma-associated herpesvirus encodes a functional cyclin. J Virol.

[52] Jarviluoma A, Koopal S, Rasanen S, Makela TP, Ojala PM (2004). KSHV viral cyclin binds to p27KIP1 in primary effusion lymphomas. Blood.

[53] Chaudhary PM, Jasmin A, Eby MT, Hood L (1999). Modulation of the NF-kappa B pathway by virally encoded death effector domains-containing proteins. Oncogene.

[54] Matta H, Sun Q, Moses G, Chaudhary PM (2003). Molecular genetic analysis of human herpes virus 8-encoded viral FLICE inhibitory protein-induced NF-kappaB activation. J Biol Chem.

[55] Sun Q, Matta H, Chaudhary PM (2003). The human herpes virus 8-encoded viral FLICE inhibitory protein protects against growth factor withdrawal-induced apoptosis via NF-kappa B activation. Blood.

[56] Sun Q, Zachariah S, Chaudhary PM (2003). The human herpes virus 8-encoded viral FLICE-inhibitory protein induces cellular transformation via NF-kappaB activation. J Biol Chem.

[57] Pfeffer S, Sewer A, Lagos-Quintana M, Sheridan R, Sander C, Grasser FA, van Dyk LF, Ho CK, Shuman S, Chien M, Russo JJ, Ju J, Randall G, Lindenbach BD, Rice CM, Simon V, Ho DD, Zavolan M, Tuschl T (2005). Identification of microRNAs of the herpesvirus family. Nat Methods.

[58] Samols MA, Hu J, Skalsky RL, Renne R (2005). Cloning and identification of a microRNA cluster within the latency-associated region of Kaposi's sarcoma-associated herpesvirus. J Virol.

[59] Cai X, Lu S, Zhang Z, Gonzalez CM, Damania B, Cullen BR (2005). Kaposi's sarcoma-associated herpesvirus expresses an array of viral microRNAs in latently infected cells. Proc Natl Acad Sci U S A.

[60] Grundhoff A, Sullivan CS, Ganem D (2006). A combined computational and microarray-based approach identifies novel microRNAs encoded by human gamma-herpesviruses. RNA.

[61] Grundhoff A, Sullivan CS (2011). Virus-encoded microRNAs. Virology.

[62] Zhu Y, Haecker I, Yang Y, Gao SJ, Renne R (2013). Gamma-herpesvirus-encoded miRNAs and their roles in viral biology and pathogenesis. Curr Opin Virol.

[63] Qin J, Li W, Gao SJ, Lu C (2017). KSHV microRNAs: tricks of the devil. Trends Microbiol.

[64] Gottwein E, Mukherjee N, Sachse C, Frenzel C, Majoros WH, Chi JT, Braich R, Manoharan M, Soutschek J, Ohler U, Cullen BR (2007). A viral microRNA functions as an orthologue of cellular miR-155. Nature.

[65] Skalsky RL, Samols MA, Plaisance KB, Boss IW, Riva A, Lopez MC, Baker HV, Renne R (2007). Kaposi's sarcoma-associated herpesvirus encodes an ortholog of miR-155. J Virol.

[66] Boss IW, Nadeau PE, Abbott JR, Yang Y, Mergia A, Renne R (2011). A Kaposi's sarcoma-associated herpesvirus-encoded ortholog of microRNA miR-155 induces human splenic B-cell expansion in NOD/LtSz-scid IL2Rgammanull mice. J Virol.

[67] Dahlke C, Maul K, Christalla T, Walz N, Schult P, Stocking C, Grundhoff A (2012). A microRNA encoded by Kaposi sarcoma-associated herpesvirus promotes B-cell expansion in vivo. PLoS One.

[68] Dittmer D, Lagunoff M, Renne R, Staskus K, Haase A, Ganem D (1998). A cluster of latently expressed genes in Kaposi's sarcoma-associated herpesvirus. J Virol.

[69] Sadler R, Wu L, Forghani B, Renne R, Zhong W, Herndier B, Ganem D (1999). A complex translational program generates multiple novel proteins from the latently expressed kaposin (K12) locus of Kaposi's sarcoma-associated herpesvirus. J Virol.

[70] Rivas C, Thlick AE, Parravicini C, Moore PS, Chang Y (2001). Kaposi's sarcoma-associated herpesvirus LANA2 is a B-cell-specific latent viral protein that inhibits p53. J Virol.

[71] Parravicini C, Chandran B, Corbellino M, Berti E, Paulli M, Moore PS, Chang Y (2000). Differential viral protein expression in Kaposi's sarcoma-associated herpesvirus-infected diseases: Kaposi's sarcoma, primary effusion lymphoma, and multicentric Castleman's disease. Am J Pathol.

[72] Katano H, Sato Y, Kurata T, Mori S, Sata T (2000). Expression and localization of human herpesvirus 8-encoded proteins in primary effusion lymphoma, Kaposi's sarcoma, and multicentric Castleman's disease. Virology.

[73] Koch S, Schulz TF (2017). Rhadinoviral interferon regulatory factor homologues. Biol Chem.

[74] Moore PS, Boshoff C, Weiss RA, Chang Y (1996). Molecular mimicry of human cytokine and cytokine response pathway genes by KSHV. Science.

[75] Cesarman E, Nador RG, Bai F, Bohenzky RA, Russo JJ, Moore PS, Chang Y, Knowles DM (1996). Kaposi's sarcoma-associated herpesvirus contains G protein-coupled receptor and cyclin D homologs which are expressed in Kaposi's sarcoma and malignant lymphoma. J Virol.

[76] Cannon M (2007). The KSHV and other human herpesviral G protein-coupled receptors. Curr Top Microbiol Immunol.

[77] Abere B, Schulz TF (2016). KSHV non-structural membrane proteins involved in the activation of intracellular signaling pathways and the pathogenesis of Kaposi's sarcoma. Curr Opin Virol.

[78] Bowser BS, DeWire SM, Damania B (2002). Transcriptional regulation of the K1 gene product of Kaposi's sarcoma-associated herpesvirus. J Virol.

[79] Bowser BS, Morris S, Song MJ, Sun R, Damania B (2006). Characterization of Kaposi's sarcoma-associated herpesvirus (KSHV) K1 promoter activation by Rta. Virology.

[80] Chandriani S, Ganem D (2010). Array-based transcript profiling and limiting-dilution reverse transcription-PCR analysis identify additional latent genes in Kaposi's sarcoma-associated herpesvirus. J Virol.

[81] Wang L, Dittmer DP, Tomlinson CC, Fakhari FD, Damania B (2006). Immortalization of primary endothelial cells by the K1 protein of Kaposi's sarcoma-associated herpesvirus. Cancer Res.

[82] Chang HH, Ganem D (2013). A unique herpesviral transcriptional program in KSHV-infected lymphatic endothelial cells leads to mTORC1 activation and rapamycin sensitivity. Cell Host Microbe.

[83] Huang C, Xu M, Zhu B (2013). Epigenetic inheritance mediated by histone lysine methylation: maintaining transcriptional states without the precise restoration of marks?. Philos Trans R Soc Lond Ser B Biol Sci.

[84] Annunziato AT (2015). The fork in the road: histone partitioning during DNA replication. Genes (Basel).

[85] Petruk S, Sedkov Y, Johnston DM, Hodgson JW, Black KL, Kovermann SK, Beck S, Canaani E, Brock HW, Mazo A (2012). TrxG and PcG proteins but not methylated histones remain associated with DNA through replication. Cell.

[86] Aranda S, Mas G, Di Croce L (2015). Regulation of gene transcription by Polycomb proteins. Sci Adv.

[87] Ramachandran S, Henikoff S (2015) Replicating nucleosomes. Sci Adv 1(7). 10.1126/sciadv.1500587 10.1126/sciadv.1500587 PMC4530793 26269799

[88] Campos EI, Stafford JM, Reinberg D (2014). Epigenetic inheritance: histone bookmarks across generations. Trends Cell Biol.

[89] Moussa HF, Bsteh D, Yelagandula R, Pribitzer C, Stecher K, Bartalska K, Michetti L, Wang J, Zepeda-Martinez JA, Elling U, Stuckey JI, James LI, Frye SV, Bell O (2019). Canonical PRC1 controls sequence-independent propagation of polycomb-mediated gene silencing. Nat Commun.

[90] Yu JR, Lee CH, Oksuz O, Stafford JM, Reinberg D (2019). PRC2 is high maintenance. Genes Dev.

[91] Laugesen A, Helin K (2014). Chromatin repressive complexes in stem cells, development, and cancer. Cell Stem Cell.

[92] Di Croce L, Helin K (2013). Transcriptional regulation by Polycomb group proteins. Nat Struct Mol Biol.

[93] Schuettengruber B, Bourbon HM, Di Croce L, Cavalli G (2017). Genome regulation by polycomb and trithorax: 70 years and counting. Cell.

[94] Gunther T, Schreiner S, Dobner T, Tessmer U, Grundhoff A (2014). Influence of ND10 components on epigenetic determinants of early KSHV latency establishment. PLoS Pathog.

[95] Gunther T, Grundhoff A (2010). The epigenetic landscape of latent Kaposi sarcoma-associated herpesvirus genomes. PLoS Pathog.

[96] Toth Z, Maglinte DT, Lee SH, Lee HR, Wong LY, Brulois KF, Lee S, Buckley JD, Laird PW, Marquez VE, Jung JU (2010). Epigenetic analysis of KSHV latent and lytic genomes. PLoS Pathog.

[97] Toth Z, Brulois K, Lee HR, Izumiya Y, Tepper C, Kung HJ, Jung JU (2013). Biphasic euchromatin-to-heterochromatin transition on the KSHV genome following de novo infection. PLoS Pathog.

[98] Gunther T, Frohlich J, Herrde C, Ohno S, Burkhardt L, Adler H, Grundhoff A (2019). A comparative epigenome analysis of gammaherpesviruses suggests cis-acting sequence features as critical mediators of rapid polycomb recruitment. PLoS Pathog.

[99] Grundhoff A, Ganem D (2004). Inefficient establishment of KSHV latency suggests an additional role for continued lytic replication in Kaposi sarcoma pathogenesis. J Clin Invest.

[100] Krishnan HH, Naranatt PP, Smith MS, Zeng L, Bloomer C, Chandran B (2004). Concurrent expression of latent and a limited number of lytic genes with immune modulation and antiapoptotic function by Kaposi's sarcoma-associated herpesvirus early during infection of primary endothelial and fibroblast cells and subsequent decline of lytic gene expression. J Virol.

[101] Kar G, Kim JK, Kolodziejczyk AA, Natarajan KN, Torlai Triglia E, Mifsud B, Elderkin S, Marioni JC, Pombo A, Teichmann SA (2017). Flipping between polycomb repressed and active transcriptional states introduces noise in gene expression. Nat Commun.

[102] Harikumar A, Meshorer E (2015). Chromatin remodeling and bivalent histone modifications in embryonic stem cells. EMBO Rep.

[103] Abere B, Mamo TM, Hartmann S, Samarina N, Hage E, Ruckert J, Hotop SK, Busche G, Schulz TF (2017). The Kaposi's sarcoma-associated herpesvirus (KSHV) non-structural membrane protein K15 is required for viral lytic replication and may represent a therapeutic target. PLoS Pathog.

[104] Sharp TV, Wang HW, Koumi A, Hollyman D, Endo Y, Ye H, Du MQ, Boshoff C (2002). K15 protein of Kaposi's sarcoma-associated herpesvirus is latently expressed and binds to HAX-1, a protein with antiapoptotic function. J Virol.

[105] Blackledge NP, Rose NR, Klose RJ (2015). Targeting polycomb systems to regulate gene expression: modifications to a complex story. Nat Rev Mol Cell Biol.

[106] Perino M, van Mierlo G, Karemaker ID, van Genesen S, Vermeulen M, Marks H, van Heeringen SJ, Veenstra GJC (2018). MTF2 recruits Polycomb repressive complex 2 by helical-shape-selective DNA binding. Nat Genet.

[107] Li H, Liefke R, Jiang J, Kurland JV, Tian W, Deng P, Zhang W, He Q, Patel DJ, Bulyk ML, Shi Y, Wang Z (2017). Polycomb-like proteins link the PRC2 complex to CpG islands. Nature.

[108] Choi J, Bachmann AL, Tauscher K, Benda C, Fierz B, Muller J (2017). DNA binding by PHF1 prolongs PRC2 residence time on chromatin and thereby promotes H3K27 methylation. Nat Struct Mol Biol.

[109] Riising EM, Comet I, Leblanc B, Wu X, Johansen JV, Helin K (2014). Gene silencing triggers polycomb repressive complex 2 recruitment to CpG islands genome wide. Mol Cell.

[110] Wu X, Johansen JV, Helin K (2013). Fbxl10/Kdm2b recruits polycomb repressive complex 1 to CpG islands and regulates H2A ubiquitylation. Mol Cell.

[111] He J, Shen L, Wan M, Taranova O, Wu H, Zhang Y (2013). Kdm2b maintains murine embryonic stem cell status by recruiting PRC1 complex to CpG islands of developmental genes. Nat Cell Biol.

[112] Blackledge NP, Farcas AM, Kondo T, King HW, McGouran JF, Hanssen LL, Ito S, Cooper S, Kondo K, Koseki Y, Ishikura T, Long HK, Sheahan TW, Brockdorff N, Kessler BM, Koseki H, Klose RJ (2014). Variant PRC1 complex-dependent H2A ubiquitylation drives PRC2 recruitment and polycomb domain formation. Cell.

[113] Farcas AM, Blackledge NP, Sudbery I, Long HK, McGouran JF, Rose NR, Lee S, Sims D, Cerase A, Sheahan TW, Koseki H, Brockdorff N, Ponting CP, Kessler BM, Klose RJ (2012). KDM2B links the polycomb repressive complex 1 (PRC1) to recognition of CpG islands. Elife.

[114] van Kruijsbergen I, Hontelez S, Veenstra GJ (2015). Recruiting polycomb to chromatin. Int J Biochem Cell Biol.

[115] Toth Z, Papp B, Brulois K, Choi YJ, Gao SJ, Jung JU (2016). LANA-mediated recruitment of host Polycomb repressive complexes onto the KSHV genome during De novo infection. PLoS Pathog.

[116] Gupta N, Thakker S, Verma SC (2016). KSHV encoded LANA recruits nucleosome assembly protein NAP1L1 for regulating viral DNA replication and transcription. Sci Rep.

[117] Thakker S, Purushothaman P, Gupta N, Challa S, Cai Q, Verma SC (2015). Kaposi's sarcoma-associated herpesvirus latency-associated nuclear antigen inhibits major histocompatibility complex class II expression by disrupting Enhanceosome assembly through binding with the regulatory factor X complex. J Virol.

[118] Hu J, Yang Y, Turner PC, Jain V, McIntyre LM, Renne R (2014). LANA binds to multiple active viral and cellular promoters and associates with the H3K4methyltransferase hSET1 complex. PLoS Pathog.

[119] Szekely L, Kiss C, Mattsson K, Kashuba E, Pokrovskaja K, Juhasz A, Holmvall P, Klein G (1999). Human herpesvirus-8-encoded LNA-1 accumulates in heterochromatin- associated nuclear bodies. J Gen Virol.

[120] Di Bartolo DL, Cannon M, Liu YF, Renne R, Chadburn A, Boshoff C, Cesarman E (2008). KSHV LANA inhibits TGF-beta signaling through epigenetic silencing of the TGF-beta type II receptor. Blood.

[121] Shamay M, Krithivas A, Zhang J, Hayward SD (2006). Recruitment of the de novo DNA methyltransferase Dnmt3a by Kaposi's sarcoma-associated herpesvirus LANA. Proc Natl Acad Sci U S A.

[122] Kim KY, Huerta SB, Izumiya C, Wang DH, Martinez A, Shevchenko B, Kung HJ, Campbell M, Izumiya Y (2013). Kaposi's sarcoma-associated herpesvirus (KSHV) latency-associated nuclear antigen regulates the KSHV epigenome by association with the histone demethylase KDM3A. J Virol.

[123] Sakakibara S, Ueda K, Nishimura K, Do E, Ohsaki E, Okuno T, Yamanishi K (2004). Accumulation of heterochromatin components on the terminal repeat sequence of Kaposi's sarcoma-associated herpesvirus mediated by the latency-associated nuclear antigen. J Virol.

[124] Lim C, Lee D, Seo T, Choi C, Choe J (2003). Latency-associated nuclear antigen of Kaposi's sarcoma-associated herpesvirus functionally interacts with heterochromatin protein 1. J Biol Chem.

[125] Sun R, Liang D, Gao Y, Lan K (2014). Kaposi's sarcoma-associated herpesvirus-encoded LANA interacts with host KAP1 to facilitate establishment of viral latency. J Virol.

[126] Stuber G, Mattsson K, Flaberg E, Kati E, Markasz L, Sheldon JA, Klein G, Schulz TF, Szekely L (2007). HHV-8 encoded LANA-1 alters the higher organization of the cell nucleus. Mol Cancer.

[127] Ottinger M, Christalla T, Nathan K, Brinkmann MM, Viejo-Borbolla A, Schulz TF (2006). Kaposi's sarcoma-associated herpesvirus LANA-1 interacts with the short variant of BRD4 and releases cells from a BRD4- and BRD2/RING3-induced G1 cell cycle arrest. J Virol.

[128] Chen HS, De Leo A, Wang Z, Kerekovic A, Hills R, Lieberman PM (2017). BET-inhibitors disrupt Rad21-dependent conformational control of KSHV latency. PLoS Pathog.

[129] Lang F, Sun Z, Pei Y, Singh RK, Jha HC, Robertson ES (2018). Shugoshin 1 is dislocated by KSHV-encoded LANA inducing aneuploidy. PLoS Pathog.

[130] He M, Zhang W, Bakken T, Schutten M, Toth Z, Jung JU, Gill P, Cannon M, Gao SJ (2012). Cancer angiogenesis induced by Kaposi sarcoma-associated herpesvirus is mediated by EZH2. Cancer Res.

[131] Lu F, Stedman W, Yousef M, Renne R, Lieberman PM (2010). Epigenetic regulation of Kaposi's sarcoma-associated herpesvirus latency by virus-encoded microRNAs that target Rta and the cellular Rbl2-DNMT pathway. J Virol.

[132] Lee HR, Li F, Choi UY, Yu HR, Aldrovandi GM, Feng P, Gao SJ, Hong YK, Jung JU (2018) Deregulation of HDAC5 by viral interferon regulatory factor 3 plays an essential role in Kaposi's sarcoma-associated Herpesvirus-induced Lymphangiogenesis. MBio 9(1). 10.1128/mBio.02217-17 10.1128/mBio.02217-17 PMC5770555 29339432

[133] Wu J, Xu Y, Mo D, Huang P, Sun R, Huang L, Pan S, Xu J (2014). Kaposi's sarcoma-associated herpesvirus (KSHV) vIL-6 promotes cell proliferation and migration by upregulating DNMT1 via STAT3 activation. PLoS One.

[134] Li W, Wang Q, Feng Q, Wang F, Yan Q, Gao SJ, Lu C (2019). Oncogenic KSHV-encoded interferon regulatory factor upregulates HMGB2 and CMPK1 expression to promote cell invasion by disrupting a complex lncRNA-OIP5-AS1/miR-218-5p network. PLoS Pathog.

[135] Rossetto CC, Pari G (2012). KSHV PAN RNA associates with demethylases UTX and JMJD3 to activate lytic replication through a physical interaction with the virus genome. PLoS Pathog.

[136] Rossetto CC, Tarrant-Elorza M, Verma S, Purushothaman P, Pari GS (2013). Regulation of viral and cellular gene expression by Kaposi's sarcoma-associated herpesvirus polyadenylated nuclear RNA. J Virol.

[137] Rossetto CC, Pari GS (2011). Kaposi's sarcoma-associated herpesvirus noncoding polyadenylated nuclear RNA interacts with virus- and host cell-encoded proteins and suppresses expression of genes involved in immune modulation. J Virol.

[138] Papp B, Motlagh N, Smindak RJ, Jin Jang S, Sharma A, Alonso JD, Toth Z (2019) Genome-wide identification of direct RTA targets reveals key host factors for Kaposi's sarcoma-associated Herpesvirus lytic reactivation. J Virol 93(5). 10.1128/JVI.01978-18 10.1128/JVI.01978-18 PMC6384073 30541837

[139] Mercier A, Arias C, Madrid AS, Holdorf MM, Ganem D (2014). Site-specific association with host and viral chromatin by Kaposi's sarcoma-associated herpesvirus LANA and its reversal during lytic reactivation. J Virol.

[140] Lu F, Tsai K, Chen HS, Wikramasinghe P, Davuluri RV, Showe L, Domsic J, Marmorstein R, Lieberman PM (2012). Identification of host-chromosome binding sites and candidate gene targets for Kaposi's sarcoma-associated herpesvirus LANA. J Virol.

[141] Hellert J, Weidner-Glunde M, Krausze J, Richter U, Adler H, Fedorov R, Pietrek M, Ruckert J, Ritter C, Schulz TF, Luhrs T (2013). A structural basis for BRD2/4-mediated host chromatin interaction and oligomer assembly of Kaposi sarcoma-associated herpesvirus and murine gammaherpesvirus LANA proteins. PLoS Pathog.

[142] Escobar TM, Kanellopoulou C, Kugler DG, Kilaru G, Nguyen CK, Nagarajan V, Bhairavabhotla RK, Northrup D, Zahr R, Burr P, Liu X, Zhao K, Sher A, Jankovic D, Zhu J, Muljo SA (2014). miR-155 activates cytokine gene expression in Th17 cells by regulating the DNA-binding protein Jarid2 to relieve polycomb-mediated repression. Immunity.

[143] Naipauer J, Rosario S, Gupta S, Premer C, Mendez-Solis O, Schlesinger M, Ponzinibbio V, Jain V, Gay L, Renne R, Chan HL, Morey L, Salyakina D, Abba M, Williams S, Hare JM, Goldschmidt-Clermont PJ, Mesri EA (2019). PDGFRA defines the mesenchymal stem cell Kaposi's sarcoma progenitors by enabling KSHV oncogenesis in an angiogenic environment. PLoS Pathog.

[144] McHugh D, Caduff N, Barros MHM, Ramer PC, Raykova A, Murer A, Landtwing V, Quast I, Styles CT, Spohn M, Fowotade A, Delecluse HJ, Papoudou-Bai A, Lee YM, Kim JM, Middeldorp J, Schulz TF, Cesarman E, Zbinden A, Capaul R, White RE, Allday MJ, Niedobitek G, Blackbourn DJ, Grundhoff A, Munz C (2017). Persistent KSHV infection increases EBV-associated tumor formation in vivo via enhanced EBV lytic gene expression. Cell Host Microbe.

